# Synthesis and Anti-Inflammatory Activity of New Alkyl-Substituted Phthalimide 1*H*-1,2,3-Triazole Derivatives

**DOI:** 10.1100/2012/925925

**Published:** 2012-12-05

**Authors:** Shalom Pôrto de Oliveira Assis, Moara Targino da Silva, Ronaldo Nascimento de Oliveira, Vera Lúcia de Menezes Lima

**Affiliations:** ^1^Laboratório de Química e Metabolismo de Lipídeos e Lipoproteínas, Departamento de Bioquímica, Universidade Federal de Pernambuco (UFPE), Avenida Prof. Moraes Rego s/n, 50670-420 Recife, PE, Brazil; ^2^Laboratório de Síntese de Compostos Bioativos (LSCB), Departamento de Ciências Moleculares, Universidade Federal Rural de Pernambuco (UFRPE), Rua Dom Manoel de Medeiros s/n, Dois Irmãos, 52171-900 Recife, PE, Brazil

## Abstract

Four new 1,2,3-triazole phthalimide derivatives with a potent anti-inflammatory activity have been synthesized in the good yields by the 1,3-dipolar cycloaddition reaction from *N*-(azido-alkyl)phthalimides and terminal alkynes. The anti-inflammatory activity was determined by injecting carrageenan through the plantar tissue of the right hind paw of Swiss white mice to produce inflammation. All the compounds **3a–c** and **5a–c** exhibited an important anti-inflammatory activity; the best activity was found for the compounds **3b** and **5c**, which showed to be able to decrease by 69% and 56.2% carrageenan-induced edema in mice. These compounds may also offer a future promise as a new anti-inflammatory agent.

## 1. Introduction

Nonsteroidal anti-inflammatory drugs (NSAIDs) are widely used for reducing pain and swellings associated with inflammation and represent an area in continuous and evergrowing development. Phthalimide derivatives are an interesting class of compounds because they possess important biological activities [1], such as anti-inflammatory [2] and hypolipidemic [3, 4] ones. Glycoconjugates play an important role in many biological processes, including cellular recognition, particularly in cases of inflammation, tumor metastasis, and immune response in bacterial and viral infections [5].

The literature describes a series of *N*-phthalimidomethyl 2,3-dideoxy- and 2,3-unsaturated sugar derivatives that showed a potent anti-inflammatory activity, by exhibiting reduction of the edema induced by carrageenan [2]. The anti-inflammatory activity has also been recently related to 1,2,3-triazole derivatives [6]. The 1,2,3-triazole is a five-membered ring structure containing three nitrogen atoms and is a ring bioisosterism case of the 1,2,4-triazole. Triazoles and derivatives display a diversity of biological activities [7], such as anti-inflammatory [6, 8], hypolipidemic [4], antimicrobial [9], anticonvulsant, [10] and anti-nociceptive [11] ones. Most of these compounds and their related activity are associated with an anti-inflammatory profile [8, 9].

**Figure 1:**
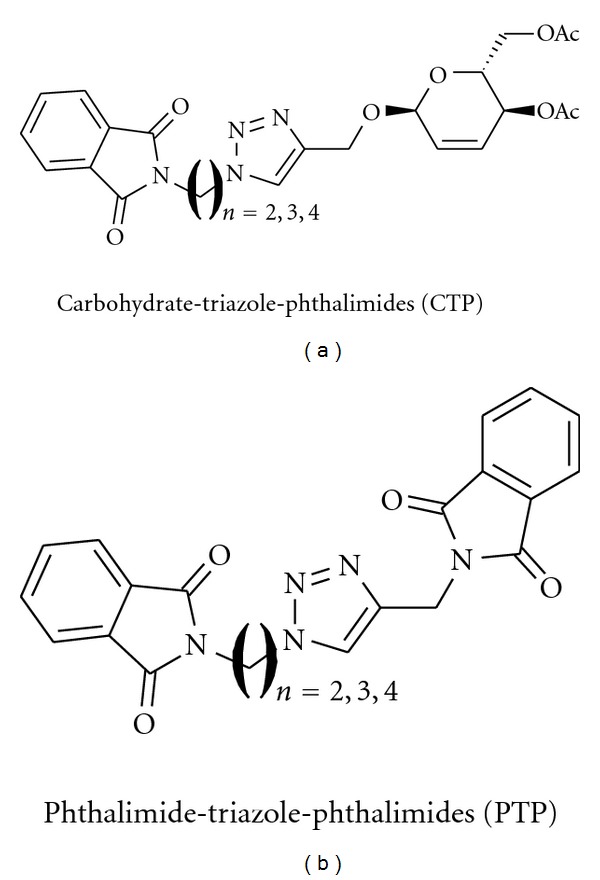
Molecules based on Carbohydrate-Triazole-Phthalimides (CTP) and Phthalimide-Triazole-Phthalimides (PTP).

1,2,3-Triazole represents a class of compounds that has awakened an interest in our research group by the synthesis of new heterocyclic derivatives and their biological evaluation [12–14]. In this work the synthesis and anti-inflammatory activity of six compounds based on 1,2,3-triazoles it were performed. One group of compounds was 1,2,3-triazoles linked to unsaturated carbohydrate and phthalimides (CTP), and another one was 1,2,3-triazoles linked to phthalimides (PTP), as shown in Figure 1. These compounds were obtained through the reaction between *N*-(azido-alkyl) phthalimides and terminal alkynes employing Cu-AAC (Cu-Catalyzed Azide Alkyne Cycloadditon) reaction [15].

## 2. Materials and Methods

### 2.1. Chemistry

All reactions were monitored by TLC analysis containing GF254 and revealed in vanillin. Melting points were determined in an open capillary tube and performed on a PFM II BioSan apparatus. Elemental analyses were carried out on an EA 1110 CHNS-O analyzer from Carlo Erba Instruments. The infrared spectra were recorded on an IFS66 Bruker spectrophotometer using KBr discs. ^1^H and ^13^C NMR were obtained on Varian Unity Plus-300 and Varian UNMRS 400 MHz spectrometer using CDCl_3_ or DMSO-d_6_ as a solvent. The polarimeter used was the Krüss, of 10 cm path length and concentration of the solution in g/100 mL. The purity of the compounds was attested on an HPLC-DAD Shimadzu Prominence Model.

#### 2.1.1. Synthesis of the Compound **1**

The compound **1** was prepared according to a reported procedure [16]. The ^1^H and ^13^C NMR data were according to the literature [17]. Yield: 82%. Mp 48°C. [*α*]_*D*^25^_ + 154 (c 0.5, CH_2_Cl_2_).

#### 2.1.2. Synthesis of the Compounds **2a–c**

The compounds **2a–c** were prepared as previously reported, and the ^1^H and ^13^C NMR data were according to the literature [12].

**N-2-(azidoethyl)phthalimide 2a**

Yield: 75%. Solid. Mp 60-61°C.

**N-3-(azidopropyl)phthalimide 2b**

Yield: 76%. Solid. Mp 40°C.

**N-4-(azidobutyl)phthalimide 2c**

Yield: 70%. Solid. Mp 45–48°C.

#### 2.1.3. Synthesis of the Compounds **3a–c**

The compound **1** (200 mg, 0.69 mmol) was transferred into 50 mL flask, and it was added to 10 mL of dichloromethane. Then, a solution containing 20 mol% copper iodide (0.0268 g, according to alkyne-compound), azide-phthalimides **2a–c** (1.2 equiv), and triethylamine (0.006 g~1 drop) was added. The mixture was stirred overnight (12 h) at r.t. (28°C) under argon atmosphere. A thin layer chromatography (TLC) was used to check the end of the reaction, using hexane : EtOAc (7 : 3) as the developing solvent system. The purification was performed by column chromatography on Merck silica gel 60 (70–230 mesh), using a system hexane : EtOAc (5 : 5). After the solvent evaporation, the product was crystallized in ethyl acetate.

**4-(4,6-di-O-acetyl-2,3-dideoxy-*α*-D-erythro-hex-2-enopyranoside)-O-methyl-1-(2-phthalimidoethyl)-1,2,3-triazole (3a)**

Yield: 90%. Solid. Mp 103–106°C. *R*_*f*_ = 0.40 (hexane : EtOAc, 3 : 7). [*α*]_*D*^25^_ + 63.3 (c 1, CH_2_Cl_2_); IR (KBr, cm^−1^): 3400, 3100, 2950, 1716, 1428, 1395, 1237, 1042, 718. ^1^H NMR (300 MHz, DMSO-d_6_): *δ* 2.02 (s, 3H, CH_3_CO), 2.06 (s, 3H, CH_3_CO), 3.94–4.03 (m, 3H, NCH_2_ and H-5), 4.11–4.16 (m, 2H, H-6 and H-6′), 4.56–4.70 (m, 4H, OCH_2_ and NCH_2_Phth), 5.09 (bs, 1H, H-1), 5.20 (dd, 1H, *J* = 9.6 and 1.5 Hz, H-4), 5.83–5.85 (m, 2H, H-2 and H-3), 7.84 (m, 4H, phthalimide), 8.18 (s, 1H, H_triazole_). ^13^C NMR (75.5 MHz, CDCl_3_): *δ* 20.8, 20.9, 37.6, 47.9, 61.2, 62.0, 65.2, 66.9, 93.4, 123.6, 127.5, 129.4, 131.6, 134.3, 167.6, 170.3, 170.8. Anal. Calc. for C_23_H_24_N_4_O_8_: C, 57.02; H, 4.99; N, 11.56. Found: C, 57.29; H, 5.36; N, 11.42.

**4-(4,6-di-O-acetyl-2,3-dideoxy-*α*-D-erythro-hex-2-enopyranoside)-O-methyl-1-(3-phthalimidopropyl)-1,2,3-triazole (3b)**

Yield: 81%. Solid. Mp 125–127°C. *R*_*f*_ 0.40 (hexane : EtOAc, 3 : 7). [*α*]_*D*^25^_ + 49.6 (c 1, CH_2_Cl_2_); IR (KBr, cm^−1^): 3468, 2925, 2854, 1770, 1710, 1491, 1399, 1231, 1037, 722. ^1^H NMR (300 MHz, DMSO-d_6_): *δ* 2.03 (s, 3H, CH_3_CO), 2.05 (s, 3H, CH_3_CO), 2.18 (q, 2H, NCH_2_), 3.61 (t, 2H, *J* = 6.9 Hz, NCH_2_), 3.98 (ddd, 1H, *J* = 9.3, 5.1 and 3.0 Hz, H-5), 4.12–4.15 (m, 2H, H-6 and H-6′), 4.42 (t, 2H, *J* = 6.9 Hz), 4.59 (d, 1H, *J* = 12 Hz, OCHa), 4.72 (d, 1H, *J* = 12.3 Hz, OCHb), 5.17 (s, 1H, H-1), 5.20 (d, 1H, *J* = 9.6 Hz, H-4), 5.86 (bs, 2H, H-2 and H-3), 7.82–7.87 (m, 4H, phthalimides), 8.12 (s, 1H, H_triazole_). ^13^C NMR (75.5 MHz, CDCl_3_): *δ* 20.8, 20.9, 29.4, 34.9, 47.2, 60.5, 62.8, 65.2, 67.0, 93.8, 122.0, 123.4, 127.5, 128.8, 129.5, 131.7, 134.3, 143.3, 168.3, 170.3, 170.9. Anal. Calc. for C_24_H_26_N_4_O_8_: C, 57.83; H, 5.26; N, 11.24. Found: C, 57.88; H, 5.46; N, 11.14.

**4-(4,6-di-O-acetyl-2,3-dideoxy-*α*-D-erythro-hex-2-enopyranoside)-O-methyl-1-(4-phthalimidobutyl)-1,2,3-triazole (3c)**

Yield: 68%. Solid. Mp 103–106°C. *R*_*f*_ 0.40 (hexane : EtOAc, 3 : 7). [*α*]_*D*^25^_ + 37.9 (c 1, CH_2_Cl_2_); IR (KBr, cm^−1^): 3464, 3141, 2935, 1769, 1711, 1437, 1398, 1370, 1230, 1038, 721. ^1^H NMR (400 MHz, DMSO-d_6_): *δ* 1.54–1.60 (q, 2H, CH_2_), 1.82–1.85 (q, 2H, CH_2_), 2.03 (s, 3H, CH_3_CO), 2.05 (s, 3H, CH_3_CO), 3.60 (t, 2H, *J* = 6.8 Hz, NCH_2_), 3.98 (ddd, 1H, *J* = 8.8, 5.2 and 3.2 Hz, H-5), 4.09–4.17 (m, 2H, H-6 and H-6′), 4.37 (t, 2H, *J* = 6.8 Hz), 4.60 (d, 1H, *J* = 12.4 Hz, OCH_a_), 4.72 (d, 1H, *J* = 12.4 Hz, OCH_b_), 5.17 (s, 1H, H-1), 5.20 (d, 1H, *J* = 9.6 Hz, H-4), 5.86 (bs, 2H, H-2 and H-3), 7.82–7.87 (m, 4H, phthalimides), 8.10 (s, 1H, H_triazole_). ^13^C NMR (75.5 MHz, CDCl_3_): *δ* 20.8, 20.9, 25.5, 27.4, 29.6, 36.7, 49.4, 61.4, 62.7, 65.1, 66.9, 93.7, 122.7, 123.2, 127.4, 129.4, 131.8, 134.0, 144.0, 168.3, 170.2, 170.8. Anal. Calc. for C_25_H_28_N_4_O_8_: C, 58.59; H, 5.51; N, 10.93. Found: C, 58.47; H, 5.30; N, 10.56.

#### 2.1.4. Synthesis of the Compounds **5a–c**

The N-(azido-alkyl)phthalimide **2a–c** was transferred into 50 mL flask and it was added to 10 mL of dichloromethane. Then, a solution of 20 mol% copper iodide (according to alkyne-compound), 1.5 equiv. N-propargyl phthalimide **4**, and triethylamine (20 mol%) was added. The mixture was stirred for 12 h at room temperature (28°C) under argon atmosphere. The purification was performed by column chromatography on Merck silica gel 60 (70–230 mesh). After the solvent evaporation, the product was crystallized in dichloromethane-hexane.

**4-(N-Phthalimidomethyl)-1-(2-phthalimidoethyl)-1,2,3-triazole (5a)**

Yield: 77%. Solid. Mp 226–230°C. *R*_*f*_ 0.14 (hexane : EtOAc, 1 : 1). IR (KBr, cm^−1^): 3582, 3459, 3144, 3094, 2951, 1771, 1703, 1466, 1428, 1398, 1322, 1224, 1101, 936, 718, 531. ^1^H NMR (400 MHz, CDCl_3_): *δ* 4.13 (t, 2H, *J* = 6.4 Hz, NCH_2_), 4.64 (t, 2H, *J* = 6.0 Hz, NCH_2_), 4.97 (s, 2H, CH_2_), 7.68–7.73 (m, 5H, phthalimide and H_triazole_), 7.78 (m, 2H, phthalimide), 7.85 (m, 2H, phthalimide). ^13^C NMR (100 MHz, CDCl_3_): *δ* 33.0, 37.6, 47.9, 123.4, 123.6, 131.7, 132.1, 134.0, 134.2, 167.6. Anal. Calc. for C_21_H_15_N5O_4_ (0.9·H_2_O): C, 60.40; H, 4.05; N, 16.77. Found: C, 60.70; H, 4.35; N, 16.49.

**4-(N-Phthalimidomethyl)-1-(3-phthalimidopropyl)-1,2,3-triazole (5b)**

Yield: 62%. Solid. Mp 150–152°C; *R*_*f*_ 0.44 (CH_2_Cl_2_ : EtOAc, 9 : 1). The NMR ^1^H and ^13^C data is in accordance with the literature [12].

**4-(N-Phthalimidomethyl)-1-(4-phthalimidobutyl)-1,2,3-triazole (5c)**

The purification started with 20% EtOAc in hexane to 100% ethyl acetate. The product was crystallized in dichloromethane and hexane. Yield: 88%. Solid. Mp 151–153°C; Lit. 150–151°C. *R*_*f*_ 0.34 (CH_2_Cl_2_ : EtOAc, 9 : 1). The spectroscopic data is in agreement with the reported data [12].

### 2.2. Pharmacology

#### 2.2.1. Animals

Three-month-old Swiss white mice, 25–30 g body weight, were maintained with water and food (Labina, Agribands Brazil Ltd.) *ad libitum*. Groups of 10 animals were separate for each experiment. All experiments reported here are in accordance with the Animal Care and Use Committee at the Federal University of Pernambuco and guidelines for Care and Use of Laboratory Animals (of. number 098/2002).

#### 2.2.2. Acute Anti-Inflammatory Activity

The drugs used for comparison purposes were **3a–c**, **5a–c**, ibuprofen, and ASA. All compounds were suspended in 1% carboxymethylcellulose (CMC) and single dose of 250 mg/Kg was administered intraperitoneally, in the morning [18]. Other animal group received 1% CMC. Two positive and one negative anti-inflammatory control tests were done in three animal groups by intraperitoneal administration of 250 mg/Kg of acetylsalicylic acid (ASA), a standard dose for pharmacological comparative tests, 250 mg/Kg of ibuprofen (Laboratory Teuto Brazilian Ltd., Brazil), and 0.9% of aqueous saline solution, respectively. The anti-inflammatory activity was determined by Levy's method [19]. Carrageenan (Sigma, St. Louis, USA), 0.1 mL of a 1% solution in 0.9% NaCl, was injected through the plantar tissue of the right hind paw of each mouse to produce inflammation. After four hours, the animals were sacrificed under anesthesia and their paws were cut and weighed. The results were analyzed according to the percentage of inflammation reduction as described earlier [19].

#### 2.2.3. Effective Dose for Anti-Inflammatory Activity

Compounds **3b** and **5c**, ASA, and ibuprofen (obtained from Bristol-Myers Squibb, Brazil) were dissolved individually in 1% CMC and administrated as described above at doses of 50, 100, 150, 200, 250, and 350 mg/Kg.

#### 2.2.4. Calculation of Octanol-Water Partition Coefficient −log⁡*P* [20]

The structures of compounds **3a–c** and **5a–c **were analyzed in ACD/Labs that contains a database available for this procedure. The values of octanol-water partition coefficient (log⁡*P*) for these compounds were predicted using Advanced Chemistry Development Inc. (ACD/Labs, algorithm version: v5.0.0.184). 

#### 2.2.5. Statistics

All results are expressed as mean ± SEM for experiments. Statistical evaluation was undertaken by analysis of variance (ANOVA) followed by Turkey test for multiple comparisons. *P* < 0.05 was used as the criterion of statistical significance.

## 3. Results and Discussion

### 3.1. Chemistry

**Scheme 1:**
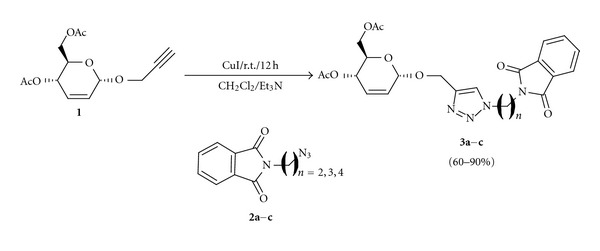
Synthesis of compounds **3a–c**.

The synthetic strategy to obtain the compounds **3a–c** consists of two convergent steps. First, the alkyne carbohydrate **1** was prepared in 82% of yield according to Ferrier's protocol [16]. In parallel, the synthesis of *N*-(azido-alkyl)phthalimides **2a–c** was achieved from the corresponding *N*-(bromoalkyl)phthalimide in good yields (70%–75%). Finally, the glycoconjugate 1,2,3-triazoles **3a–c** were initially prepared using the protocol described in a previous work [12] to afford the compound **3a** in moderate yield (48%). Thus, when 1 drop of triethylamine was added the desired compounds were obtained in good yield (90%). Under this optimized conditions the compounds **3a–c** were synthesized in good yields of 68%–90% after column chromatography on silica gel (Scheme 1). 

**Scheme 2:**
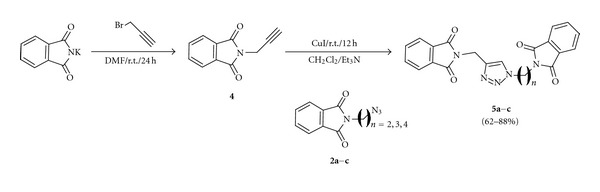
Synthesis of compounds** 5a–c**.

The *N*-propargyl phthalimide **4** was prepared from potassium phthalimide and propargyl bromide after being stirred at room temperature for 24 h. The reaction between alkyne **4** and *N*-(azido-alkyl)-phthalimides **2a–c** was carried out at the same conditions described above. This facile protocol provided the bis-phthalimides 1,2,3-triazole **5a–c** in good yields of 62%–88%, as shown in Scheme 2.

### 3.2. Pharmacology

All the compounds exhibited anti-inflammatory activity when compared with ASA as well as ibuprofen. The compounds **3a**, **3b**, and **3c** reduced carrageenan-induced edema in Swiss white mice by 33.7%, 69%, and 44%, respectively. This result is significant (*P* < 0.001) when compared with the control group treated with the saline solution (Table 1). 

The compounds **5a**,** 5b**, and** 5c** were able to reduce the edema by 17%, 25%, and 56.2%, respectively, as shown in Table 1. Therefore, substances **3b** and **5c **showed the best anti-inflammatory activity in terms of edema inhibition. 

**Figure 2:**
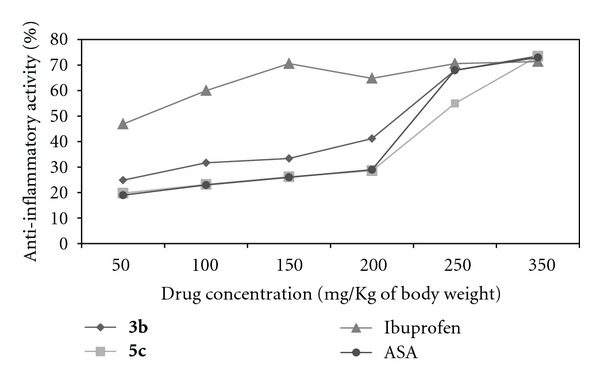
Anti-inflammatory dose-response curves for compounds **3b**, **5c**, ASA, and ibuprofen using carrageenan to produce inflammation in the paw of mice.

The **3b** and **5c **compounds, which showed the greatest anti-inflammatory effect, were selected for the experiment of determining the effective dose. The study of effective dose has shown that closely at concentration of 225 mg/Kg the compounds **3b** and **5c** have 50% of anti-inflammatory activity (Figure 2). The results of anti-inflammatory dose-response curves for the compounds **3b** and **5c** showed similar results to the ASA at all concentrations tested. On the other hand, the anti-inflammatory effect of compounds **3b** and **5c** was similar to ibuprofen only when administered at high dose (350 mg/Kg), as shown in Figure 2. 

**Figure 3:**
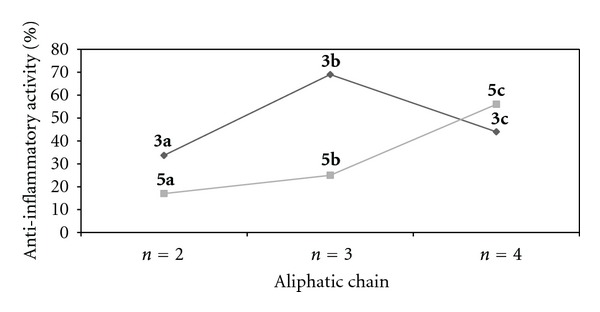
Analysis of anti-inflammatory activity (%) versus variation of size aliphatic chain in the compounds **3a–c **(CTP) and **5a–c **(PTP).

According to Table 1 the acute anti-inflammatory activity of considering the compounds **3a–c** containing phthalimide linked to carbohydrate (CTP) the best anti-inflammatory activity was observed for the compound **3b** that contains three methylene groups. However, for compounds **5a–c** that have two moieties of phthalimide connected to a triazole ring (PTP), when increasing the size of aliphatic chain, the anti-inflammatory activity is also increased from 17% (*n* = 2) to 56.2% (*n* = 4) (Table 1 and Figure 3). 

Hydrophobic interactions play a key role in the folding and maintenance of the three-dimensional structure of proteins, as well as in the binding of ligands (e.g., drugs) to protein targets [21]. Protein-ligand binding is partially driven by lipophilic interaction, and the log⁡*P* for a class of ligand compounds will depend on the nature of protein. In fact, it is known in the literature that chain branching or homologation can cause the molecule to bind more or less well to the receptors responsible for specific biological activity [22]. 

In order to evaluate the chain effect on the anti-inflammatory activity we have calculated the partition coefficient (log⁡*P*) using the ADC/Labs PhysChem Predictor. The log⁡*P* values are important data to be considered for drug design and their pharmacokinetics properties; they indicate the lipophilic tendency or the ability to penetrate lipid barriers (lipophilicity) [23]. 

The chain effect for the biological activity in both classes of the compounds **3** and **5 **is shown in Table 2, and the log⁡*P* ranged from 1.53 to 2.04 and from 2.37 to 2.89, respectively. 

The compounds **3a–c** have a portion carbohydrate, and for this class we can note that a peak anti-inflammatory activity occurred with the 3-phthalimide propyl-1,2,3-triazole **3b **(Figure 3). On the other hand, the class of compounds **5a–c** is more lipophilic and showed to be sensitive to anti-inflammatory activity when the methylene groups were introduced. 

In recent works, a new series of 1-[2-(1*H*-tetrazol-5-yl)ethyl]-1*H*-benzo[d][1,2, 3]triazoles have shown anti-inflammatory activity (11–47%) against carrageenan-induced paw edema, whereas the standard drug diclofenac sodium showed 61% [24]. Other novel series of azoles, such as pyrazole, were obtained by reacting chalcones and hydrazine hydrate, and they exhibited low inhibition of paw edema ranging 1.08–31.05%, and these results corresponded to a percentage of the indomethacin inhibition [25]. However, in the present work, the compounds **3b** and **5c** exhibited better anti-inflammatory activity (Figure 2) than that reported by Khalil [25], when considering the anti-inflammatory effect of **3b** and **5c** observed with 50 mg/Kg (lower dose) in relation to the same concentration of ibuprofen or ASA instead of that of the saline.

Recently, a series of new 1,2,4-triazoles obtained by reacting acyl 1,2,4-triazoles with various secondary amines, exhibited appreciable inhibition [8]; when, administered at a dose of 100 mg/Kg, the percentage inhibition reached 55.6%, the comparison was performed with the reference drug indomethacin (62.5% at 100 mg/Kg). These results are similar to the compounds **3b** and **5c**, which were administered by us at a dose of 250 mg/Kg.

There are scientific evidences that the *bis*-heterocyclic compounds encompassing 2-mercapto benzothiazole and 1,2,3-triazole showed significant binding potential towards COX enzyme thus lowering the paw edema induced by carrageenan [6]. The class of monoacylated 5-amino-1,2,4-triazole derivatives exhibited potent anti-inflammatory activity (at 5 mg/kg, oral dose level) in carrageenan-induced rat paw edema test [26]. These results also contributed to show that many structure containing the heterocyclic triazole possessed high anti-inflammatory activity.

New 1H-(1,2,3-triazole)phthalimide derivatives are interesting drugs due to their potential anti-inflammatory activity, thus deserving further studies in order to understand the mechanism of action. In conclusion, the results suggest that these compounds may also offer a future promise as a new anti-inflammatory agent.

## Figures and Tables

**Table 1:** Acute anti-inflammatory activity of 1,2,3-triazole phthalimides **3a–c** and **5a–c** after carrageenin-induced edema at dose 250 mg·kg^−1^.

Compounds	Difference in paw weight (**g**) ± SEM	Edema inhibition (%)
**3a**	0.1030 ± 0.0129**	33.7
**3b**	0.0485 ± 0.0160**	69.0
**3c**	0.0865 ± 0.0385**	44.0
**5a**	0.1247 ± 0.0119**	17.0
**5b**	0.1166 ± 0.0308*	25.0
**5c**	0.0680 ± 0.0128**	56.2
1% Carboxymethylcellulose	0.1461 ± 0.0168^ns^	6.0
0.9% saline solution	0.1553 ± 0.0172^ns^	—
Ibuprofen	0.0421 ± 0.0134**	73.0
ASA	0.0502 ± 0.0237**	68.0

Significant differences: *P < 0.05; **P < 0.001; ns: not significant.

**Table 2:** Structure activity and log⁡*P* values calculated.

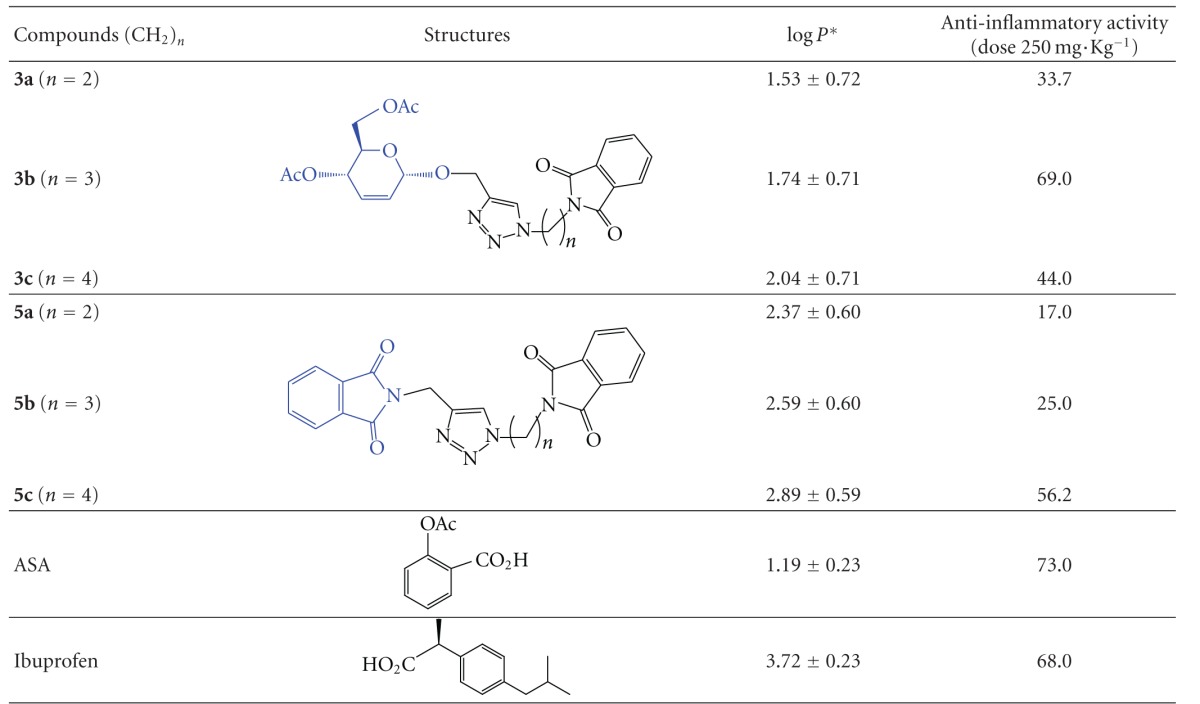

*log⁡*P* values were calculated using Advanced Chemistry Development (ACD/Labs Algorithm Version: v5.0.0.184).
